# Superconductivity in Empty Bands and Multiple Order Parameter Chirality

**DOI:** 10.1038/s41598-017-13426-9

**Published:** 2017-10-11

**Authors:** Robert Joynt, Wen-Chin Wu

**Affiliations:** 1 Department of Physics, University of Wisconsin-Madison, Madison, WI 53706 USA; 2 International Center for Quantum Materials, School of Physics, Peking University, Beijing, 100871 China; 3 Department of Physics, National Taiwan Normal University, Taipei, 11677 Taiwan

## Abstract

Recent experiments have shown rotation of the plane of polarization of light reflected from the surface of some superconductors. The photon energy exceeds the electronic bandwidth, so that completely filled or completely empty bands must play a role. We show that in strong-coupling theory a Coulomb interaction can produce an order parameter in the unoccupied band that explains the observations. Thus the phenomenology puts tight constraints on the form of the order parameter in different bands. We propose that the experiments have detected, for the first time, the existence of a superconducting order parameter in a band far from the Fermi energy. This is only possible because of the sensitivity to delicate symmetries: a positive Kerr effect indicates that time reversal and certain mirror symmetries are broken in the ordered phase. Furthermore, detailed analysis of the results implies that in UPt_3_ there exist bands that have different order parameter chiralities, opening up complex new possibilities for topological superconductivity.

## Introduction

The subject of unconventional superconductivity is now over 30 years old, and the prime driving force in the field has been the determination of the form of the order parameter. This is important in itself for possible applications in quantum computing, and also in order to determine the mechanism of superconductivity. This issue has become more urgent in the era of topological superconductivity. The determination has generally proved to be surprisingly difficult: unambiguous identifications remain remarkably few in number.

Experiments at optical frequencies are rarely useful in this connection. Superconductivity is fundamentally a low-frequency phenomenon, and modification of interband matrix elements by superconductivity are miniscule. The Kerr effect^1^, rotation of the plane of polarization of reflected light through an angle *θ*_*K*_, is an important possible exception to this venerable rule, since it tests time-reversal symmetry breaking.

**Figure 1:**
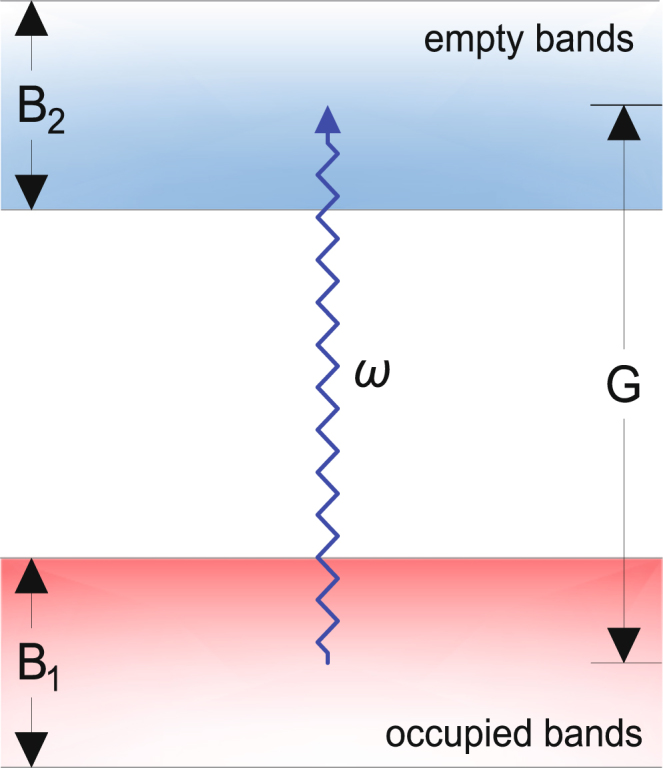
Schematic plot showing the energy scales in the Kerr rotation angle experiment on UPt_3_^6^. The photon energy *ħω* = 0.8 eV is about the energy separation between occupied and empty bands *G*, while the band widths of the occupied and empty bands *B*_1_ and *B*_2_ ≈ 0.2 eV, which is smaller than *ħω*. Thus absorption of the light and the consequent polarization rotation is due to interband transitions.

The Kerr effect in superconductors was already an active area of research both in experiments^2^ and theories^3,4^ in the 1990s. However, positive experimental results are relatively recent. A nonzero signal that begins at the onset of superconductivity and grows as temperature decreases has been observed in Sr_2_RuO_4_^5^, UPt_3_^6^, and URu_2_Si_2_^7^. The simplest theories of the pure system do not give a result large enough to explain the observed magnitude of *θ*_*K*_ ≈ 10^−6^. In Sr_2_RuO_4_ this has been interpreted in different ways. One is to invoke impurity scattering^8–10^. Another is to attribute the effect to interband transitions between bands that cross the Fermi energy^11,12^. This paper focuses on clean UPt_3_, though we comment on other compounds below. This is the simplest case for our purposes, since in UPt_3_ the bandwidth is less than the energy (0.8 eV) of the light^13,14^ (see Fig. 1 for a schematic plot). Thus the transitions induced by the light can only connect partially occupied with completely unoccupied bands and we have a clear-cut example of a material for which the above theories can be ruled out.

We propose that the Coulomb interaction induces a nonzero order parameter in the unoccupied bands that is responsible for the Kerr effect. This implies that the Kerr effect experiments probe, for the first time, a superconducting order parameter in an unoccupied band distant from the Fermi energy. A somewhat similar effect was proposed for semiconductors with small gaps^15^ and may have been seen in a completely occupied band relatively near the Fermi energy in LiFeAs^16^. But this would be the first time that the order parameter in a distant band has been seen in experiments. Furthermore, since the present treatment is purely phenomenological the conclusions are independent of the microscopic mechanism of superconductivity.

## Kerr Effect in Superconductors

Reflection of light of frequency *ω* incident on a sample along the *z*-axis is controlled by the components *ε*_*xx*_(*ω*), *ε*_*yy*_(*ω*), *ε*_*xy*_(*ω*), and *ε*_*yx*_(*ω*) of the dielectric tensor. UPt_3_ is hexagonal and we can assume that tetragonal distortions that give rise to the inequality of *ε*_*xx*_(*ω*) and *ε*_*yy*_(*ω*) (linear birefringence) are absent so *ε*_*xx*_(*ω*) = *ε*_*yy*_(*ω*) = *ε*. In any case, the experiments are designed not to be sensitive to linear birefringence^1^. In a field $\overrightarrow{u}$ that breaks time reversal symmetry, including an effective field from an order parameter, the Onsager relation is ${\varepsilon }_{xy}(\omega ,\overrightarrow{u})={\varepsilon }_{yx}(\omega ,-\overrightarrow{u})$ ^17^ and since the assumption of a linear relation between *ε* and $\overrightarrow{u}$ is well-founded in the present case, we find *ε*_*xy*_(*ω*) = −*ε*_*yx*_(*ω*). Then the normal modes in the metal for a propagation direction $\overrightarrow{k}=k\hat{z}$ are circularly polarized. For each frequency there are two wavevectors *k*_+_ and *k*_−_ that correspond to the two helicities: ${k}_{\pm }^{2}=({\omega }^{2}/{c}^{2}){\varepsilon }_{\pm }$ and *ε*_±_(*ω*) = *ε*_*xx*_(*ω*) ± *i*|*ε*_*xy*_(*ω*)|. The different dispersion and absorption for *k*_+_ and *k*_−_ gives the Kerr rotation ${\theta }_{K}={\rm{Re}}({\varepsilon }_{xy}/{\varepsilon }_{xx}^{\mathrm{3/2}})$ in a metal (for which |*ε*_*xx*_| $\gg$ 1). To have *ε*_*xy*_ ≠ 0 or *ε*_*yx*_ ≠ 0, we need breaking of both mirror symmetries *x* → −*x* and *y* → −*y* and time-reversal^18^. For a spatially uniform conventional singlet superconductor with order parameter Δ, time-reversal means Δ → Δ* and a change of gauge is Δ → Δ*e* ^*iϕ*^, so any time-reversal transformation is equivalent to a gauge transformation and as a result there is no non-trivial notion of time-reversal. For an unconventional superconductor with a momentum-dependent order parameter, this is not the case: we might have Δ(**p**) = *c*Δ_0_ *p*_*z*_(*p*_*x*_ + *ip*_*y*_). Then Δ*(**p**) = *c*Δ_0_ *p*_*z*_(*p*_*x*_ − *ip*_*y*_) and no uniform phase factor relates Δ and Δ*.

## Model Hamiltonian

An appropriate model Hamiltonian for the multiband superconductor UPt_3_ is:

1 $$\begin{array}{rcl}H-\mu N & = & \sum _{n,\overrightarrow{p},\sigma }\,\xi \,(n,\overrightarrow{p})\,{a}_{\sigma }^{\dagger }(n,\overrightarrow{p})\,{a}_{\sigma }\,(n,\overrightarrow{p})\\  &  & +\sum _{n,\overrightarrow{p}}\,\sum _{n^{\prime} ,\overrightarrow{p}^{\prime} }\,V\,(n,\overrightarrow{p};n^{\prime} ,\overrightarrow{p}^{\prime} )\,{a}_{\uparrow }^{\dagger }\,(n,\overrightarrow{p})\,{a}_{\downarrow }^{\dagger }\,(n,-\overrightarrow{p})\,{a}_{\downarrow }\,(n^{\prime} ,-\overrightarrow{p}^{\prime} )\,{a}_{\uparrow }(n^{\prime} ,\overrightarrow{p}^{\prime} ),\end{array}$$

where $\xi (n,\overrightarrow{p})$ are the single-particle energies measured relative to the chemical potential, *σ* is the pseudospin, *n* and *n*′ are band indices, ${a}_{\sigma }^{\dagger }(n,\overrightarrow{p})$ creates an electron in the state $n\overrightarrow{p}\sigma$, and $V(n,\overrightarrow{p};n^{\prime} ,\overrightarrow{p}^{\prime} )$ is a singlet pairing interaction. The sum runs over all bands within 0.8 eV of the Fermi energy. Since the bandwidths are of order *B* ≈ 0.2 to 0.3 eV, this includes completely full and completely empty bands as well as the usual partially occupied bands. Because of the narrow bandwidth, there is no pair of partially occupied bands that have energies as much as 0.8 eV apart.

We have restricted our model to give singlet pairing only for ease of presentation. The conclusions are essentially the same for triplet pairing. *H* is treated in the mean field approximation in a straightforward generalization of the usual BCS-Gor’kov procedure^19^. However, we include strong coupling in that we make no assumptions about frequency cutoffs for the function $V(n,\overrightarrow{p};n^{\prime} ,\overrightarrow{p}^{\prime} )$. This leads to a set of coupled gap equations

2 $${\rm{\Delta }}\,(n,\overrightarrow{p})=-\sum _{n^{\prime} \overrightarrow{p}^{\prime} }\,F\,(n^{\prime} ,\overrightarrow{p}^{\prime} )\,V\,(n,\overrightarrow{p};n^{\prime} ,\overrightarrow{p}^{\prime} )\,{\rm{\Delta }}\,(n^{\prime} ,\overrightarrow{p}^{\prime} ),$$

where $F(n,\overrightarrow{p})=\tanh \,[\beta E(n,\overrightarrow{p}\mathrm{)/2}]/[2E(n,\overrightarrow{p})]$, $E(n,\overrightarrow{p})={[{\xi }^{2}(n,\overrightarrow{p})+{|{\rm{\Delta }}(n,\overrightarrow{p})|}^{2}]}^{\mathrm{1/2}}$, and *β* is the inverse temperature. Since the experiments are done near the critical temperature, we linearize these equations with respect to Δ and $F(n,\overrightarrow{p})=\tanh \,[\beta \xi (n,\overrightarrow{p})/2]/[2\xi (n,\overrightarrow{p})]$. In this case $F(n,\overrightarrow{p})$ has the full symmetry of the lattice.

The point group of the system is *D*_6*h*_ for UPt_3_. The case of interest is that of unconventional superconductivity. Let *R* be a group operation not the identity. We have that ${\rm{\Delta }}(n,R\overrightarrow{p})\ne {\rm{\Delta }}(n,\overrightarrow{p})$ for all *n*. It is also true that $V(n,R\overrightarrow{p};n^{\prime} ,R\overrightarrow{p}^{\prime} )=V(n,\overrightarrow{p};n^{\prime} ,\overrightarrow{p}^{\prime} )$ so *V* can be decomposed into channels corresponding to the irreducible representations of *G*. Regarded as a function of $\overrightarrow{p}$, we seek the highest eigenvalue of *V*, which then determines the representation actually realized. Calculations for UPt_3_ using experimental data to estimate $V(n,\overrightarrow{p};n^{\prime} ,\overrightarrow{p}^{\prime} )$ were done years ago, but were not conclusive^20,21^ and first principles calculations using the functional renormalization group have been done for other systems^22^, but not for UPt_3_. In any case, the conclusions of this paper are independent of the microscopic model.

The split transition in UPt_3_^23,24^ implies that this representation is multi-dimensional, which for singlet superconductivity means *E*_1*g*_ or *E*_2*g*_. We choose the former for definiteness, but our conclusions apply equally to these two representations. It is important to note that in the linear regime, Eq. (2) determines the representation, but not which combination of basis functions is chosen by the system. This degeneracy is broken at higher order and there must be complex coefficients for a Kerr rotation to occur. Thus we have that ${\rm{\Delta }}(n,\overrightarrow{p})={{\rm{\Delta }}}_{0}(n,\overrightarrow{p})\,{p}_{z}({p}_{x}\pm i{p}_{y})$, where ${{\rm{\Delta }}}_{0}(n,R\overrightarrow{p})={{\rm{\Delta }}}_{0}(n,\overrightarrow{p})$ for all *R*.

## Order Parameter Symmetry in Different Bands

We may separate the bands into partially filled bands, of which there are 5 in UPt_3_ indexed by 1 ≤ *n* ≤ 5 and completely filled or empty bands, indexed by *n* > 5. There are 6 separate Fermi surfaces in UPt_3_. For *n* > 5, $F(n,\overrightarrow{p})$ is of order 1/*B* or less. We expect $|{\rm{\Delta }}(n^{\prime} ,\overrightarrow{p})|$ for *n*′ > 5 to be induced by a coupling $V(n,\overrightarrow{p};n^{\prime} ,\overrightarrow{p}^{\prime} )$ that is off-diagonal in the band indices, connecting partially filled to completely filled or completely empty bands.

Then there are two questions that are crucial for the calculation of *θ*_*K*_. 1. How are the Ising-like variables ± in the equation ${\rm{\Delta }}(n,\overrightarrow{p})={{\rm{\Delta }}}_{0}(n,\overrightarrow{p})\,{p}_{z}({p}_{x}\pm i{p}_{y})$ determined as *n* varies? 2. What is the order of magnitude of $|{\rm{\Delta }}(n,\overrightarrow{p})|$ for *n* > 5?

**Figure 2:**
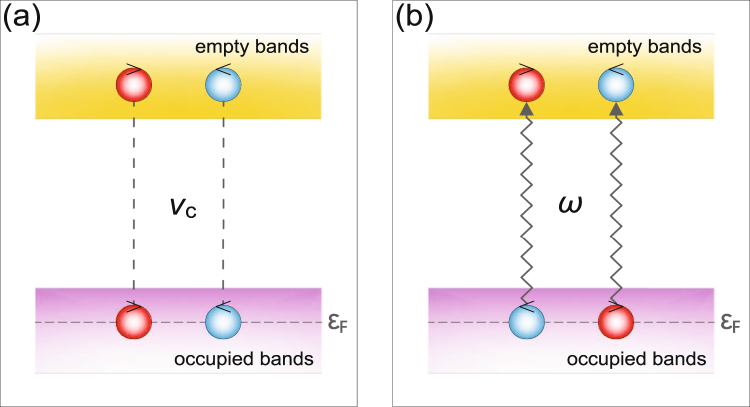
(**a**) Schematic plot showing that strong Coulomb interaction *V*_*c*_ can generate superconducting order parameters in the empty bands. Blue (red) circles denote Cooper pairs with positive (negative) chirality. Note that the chirality is interpreted as spin, and the interaction can promote chirality ferromagnetism or chirality antiferromagnetism. (**b**) Schematic plot showing that Kerr rotation creates a broken pair with different chirality of the two order parameters involved. If only one chirality is involved, then no Kerr rotation results.

1. The first question is fairly easy to answer in our model. $V(n,\overrightarrow{p};n^{\prime} ,\overrightarrow{p}^{\prime} )$ couples only bands with a (*p*_*x*_ + *ip*_*y*_) with other bands with a (*p*_*x*_ + *ip*_*y*_) gap and couples only bands with a (*p*_*x*_ − *ip*_*y*_) with other bands with a (*p*_*x*_ − *ip*_*y*_) gap, *i*.*e*., it is diagonal in the ± degree of freedom. However, this coupling can be of either sign. Of course there are no symmetries in the band index, so the couplings have no particular relation to each other. In a Ginzburg-Landau approach, we may define ${\rm{\Delta }}(n,\overrightarrow{p})={{\rm{\Delta }}}_{0}(n,\overrightarrow{p})\,{p}_{z}({\eta }_{x}{p}_{x}+{\eta }_{y}{p}_{y})$ where the “internal” order parameter $\overrightarrow{\eta }=({\eta }_{x},{\eta }_{y})$ depends on the band index. The free energy in *E*_1*g*_ is then
  3 $$F={\alpha }_{m}\,{\overrightarrow{\eta }}_{m}\cdot {\overrightarrow{\eta }}_{m}^{\ast }+{\beta }_{m}\,{({\overrightarrow{\eta }}_{m}\cdot {\overrightarrow{\eta }}_{m}^{\ast })}^{2}+{\gamma }_{m}\,{|{\overrightarrow{\eta }}_{m}\cdot {\overrightarrow{\eta }}_{m}|}^{2}+{J}_{mn}\,({\overrightarrow{\eta }}_{m}\cdot {\overrightarrow{\eta }}_{n}^{\ast }+{\overrightarrow{\eta }}_{m}^{\ast }\cdot {\overrightarrow{\eta }}_{n}),$$
  with a summation convention over the band indices *m* and *n* in effect. To break time-reversal symmetry we need the *γ*_*m*_ to be positive, and we need some of the *J*_*mn*_ to be positive for some pair (*m*, *n*) of bands that differ in energy by 0.8 eV. Then we have a problem of determining the ground state of an Ising magnet with more-or-less random couplings. We may expect both (*p*_*x*_ + *ip*_*y*_) and (*p*_*x*_ − *ip*_*y*_) to occur in the absence of physical considerations to the contrary.
2. The second question is more complicated. The size of $|{\rm{\Delta }}(n,\overrightarrow{p})|$ for the partially occupied bands (*n* ≤ 5) is at least partially constrained by experiment. We expect at least one and perhaps more of the gaps to be of order 2*k*_*B*_ *T*, *i*.*e*., about 10^−4^ eV. The superconductivity for *n* > 5 is induced from the partially occupied bands. For estimation purposes, we choose 2 bands from Eq. (2), denoting them by *g* for partially occupied and *e* for empty. We consider the separable forms: $V(g,\overrightarrow{p};g,\overrightarrow{p}^{\prime} )=-{g}_{g}f(\overrightarrow{p}){f}^{\ast }(\overrightarrow{p}^{\prime} )$ and $V(g,\overrightarrow{p};e,\overrightarrow{p}^{\prime} )=-{g}_{e}\,f(\overrightarrow{p}){f}^{\ast }(\overrightarrow{p}^{\prime} )$ with $f(\overrightarrow{p})={p}_{z}({p}_{x}+i{p}_{y})$, so that ${\rm{\Delta }}(g,\overrightarrow{p})={{\rm{\Delta }}}_{g}^{(0)}f(\overrightarrow{p})$ and ${\rm{\Delta }}(e,\overrightarrow{p})={{\rm{\Delta }}}_{e}^{(0)}f(\overrightarrow{p})$. The assumption that ${{\rm{\Delta }}}_{e}^{(0)}$ is induced means that the corresponding component of $V(e,\overrightarrow{p};e,\overrightarrow{p}^{\prime} )$ is small and we set it to zero. Then Eq. (2) yields

4 $${{\rm{\Delta }}}_{g}^{(0)}\,[1-{g}_{g}{F}_{g}({{\rm{\Delta }}}_{g}^{(0)})]={{\rm{\Delta }}}_{e}^{(0)}{g}_{e}\,{F}_{e}({{\rm{\Delta }}}_{e}^{(0)});\,{{\rm{\Delta }}}_{e}^{(0)}={{\rm{\Delta }}}_{g}^{(0)}{g}_{e}\,{F}_{g}({{\rm{\Delta }}}_{g}^{(0)}),$$

where ${F}_{g}({{\rm{\Delta }}}_{g}^{(0)})={\sum }_{\overrightarrow{p}}\,F(g,\overrightarrow{p}){|f(\overrightarrow{p})|}^{2}$ and ${F}_{e}({{\rm{\Delta }}}_{e}^{(0)})={\sum }_{\overrightarrow{p}}\,F(e,\overrightarrow{p}){|f(\overrightarrow{p})|}^{2}$. At *T* = 0 we have that ${F}_{g}({{\rm{\Delta }}}_{g}^{(0)})\approx {N}_{g}(0)\,$ $\mathrm{ln}\,({\omega }_{c}/{{\rm{\Delta }}}_{g}^{(0)})$ while ${F}_{e}({{\rm{\Delta }}}_{e}^{(0)})$ is of order 1/*ω*_*c*_. The latter estimate also requires that the cutoff *ω*_*c*_ is not too much less than the bandwidth, justified if the interaction comes from the Coulomb interaction. We find that $|{{\rm{\Delta }}}_{e}^{(0)}/{{\rm{\Delta }}}_{g}^{(0)}|\sim |{g}_{e}/{g}_{g}|$. Since all the bands are *f*-like in UPt_3_, the Coulomb matrix elements at short distances are expected to be comparable, and this gives reason to suppose that $|{{\rm{\Delta }}}_{e}^{(0)}/{{\rm{\Delta }}}_{g}^{(0)}|$ is of order unity. Figure 2(a) gives a schematic plot showing that strong Coulomb interaction *V*_*c*_ can generate superconducting order parameters in the empty bands.

## Magnitude of ***θ***_***K***_

In order to calculate *θ*_*K*_ we need the diagonal complex dielectric function *ε*_*xx*_(*ω* = 0.8 eV). This has been determined by reflectivity measurements and a Kramers-Kronig analysis^25,26^. In this frequency range it is necessary to include several Lorentz oscillators to fit the data, showing that there are interband transitions at *ω* = 0.8 eV. This is in agreement with band calculations^13,14^. We extract the approximate values Re*ε*_*xx*_(*ω* = 0.8 eV) ≈ 3 and Im*ε*_*xx*_(*ω* = 0.8 eV) ≈ 25 from these results.

The key quantity is of course the off-diagonal dielectric function *ε*_*xy*_(*ω* = 0.8 eV). The result for a single pair of bands (*m*, *n*) is

5 $${\rm{Re}}{\varepsilon }_{xy}(\omega )=\tfrac{64\pi }{{\omega }^{2}}\,\sum _{\overrightarrow{p}}\,{J}_{x}^{(mn)}\,(\overrightarrow{p})\,{J}_{y}^{(\,mn)}\,(-\overrightarrow{p})\,\tfrac{{\rm{Im}}[{\rm{\Delta }}\,(m,\overrightarrow{p}\,)\,{{\rm{\Delta }}}^{\ast }\,(n,\overrightarrow{p})]}{{E}_{m}\,(\overrightarrow{p})\,{E}_{n}\,(\overrightarrow{p})}\,\delta [\omega -{E}_{n}\,(\overrightarrow{p})-{E}_{m}\,(\overrightarrow{p})],$$

obtained from the anomalous part of the lowest-order bubble diagram. This is not the total dielectric function. To get that we must also sum over all pairs. Here ${J}_{x,y}^{(mn)}(\overrightarrow{p})$ are the interband matrix elements of the current operator between single-particle states in the partially occupied *m*-band and the empty *n*-band. An analogous expression would hold for transitions from a completely full band to a partially occupied band. This expression for *ε*_*xy*_ is to be compared to that for the familiar normal-state dielectric function

6 $${\rm{Im}}{\varepsilon }_{xx}(\omega )=\frac{32\pi }{{\omega }^{2}}\,\sum _{\overrightarrow{p}}\,{J}_{x}^{(mn)}\,(\overrightarrow{p})\,{J}_{x}^{(mn)}\,(\overrightarrow{p})\,\delta \,[\omega -{\xi }_{n}\,(\overrightarrow{p})+{\xi }_{m}\,(\overrightarrow{p})].$$

Although Eqs (5) and (6) contain quantities which are poorly known, only the ratio is involved in the Kerr angle *θ*_*K*_. It is only this that allows us to give an order of magnitude estimate for *θ*_*K*_. To make the estimate we consider two scenarios for the band structure.

*Scenario 1*. We take the single-particle energies ${\xi }_{m}(\overrightarrow{p})$, ${\xi }_{n}(\overrightarrow{p})$ to be random uncorrelated variables that are uniformly distributed over a bandwidth *B*, and the center of the *m* and *n* bands are separated by an energy *G* (see Fig. 1). In the model the averages over the current matrix elements are assumed to be the same for the two bands, and there are no correlations in momentum space between the gap functions ${\rm{\Delta }}(m,\overrightarrow{p})$, ${{\rm{\Delta }}}^{\ast }(n,\overrightarrow{p})$ and ${J}_{x}^{(mn)}(\overrightarrow{p})$, ${J}_{y}^{(mn)}(\overrightarrow{p})$. The computation of the ratio then reduces to a determination of the ratio of the density of states parts of Eqs (5) and (6). The result is:

7 $$\frac{{\rm{Re}}{\varepsilon }_{xy}}{{\rm{Im}}{\varepsilon }_{xx}}\sim \frac{2{{\rm{\Delta }}}_{m}{{\rm{\Delta }}}_{n}}{\omega B}\,\mathrm{ln}\,(\frac{{\omega }_{c}}{{{\rm{\Delta }}}_{m}})\,{I}_{s}\approx 5\times {10}^{-7}{I}_{s}.$$

Here Δ_*m*_, Δ_*n*_ are the average values of $|{\rm{\Delta }}(m,\overrightarrow{p})|$, $|{\rm{\Delta }}(n,\overrightarrow{p})|$, taken to be approximately equal to 2*k*_*B*_ *T*_*c*_, *ω*_*c*_ is the cutoff for the gap ${\rm{\Delta }}(m,\overrightarrow{p})$ and we have assumed that *ω*_*c*_ does not differ by orders of magnitude from *B*, which we take to be *B* = 0.2 eV. *I*_*s*_ is the normalized angular integral over the anisotropic gap functions. We discuss it further below. Eq. (7) gives the contribution to the ratio Re*ε*_*xy*_/Im*ε*_*xx*_ from one pair of bands. If we sum over all bands and the ratio does not vary much from pair to pair, then we may combine this value with the normal-state experimental value of *ε*_*xx*_(*ω*) quoted above to find *θ*_*K*_ ~ 2 × 10^−7^ at zero temperature, which is about 20% or so of the value one would get if the experimental results measured near *T*_*c*_ are extrapolated to *T* = 0.

**Figure 3:**
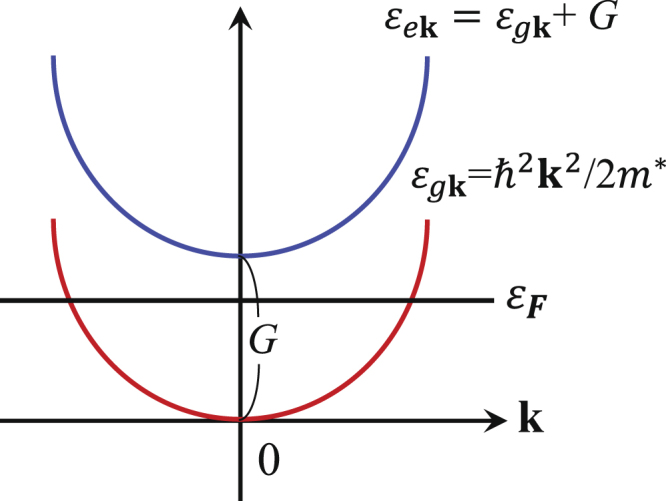
The band structure of Scenario 2. *G* denotes the average band gap between two bands. In this case the system can take advantage of a resonance and there is enhancement of the Kerr rotation.

*Scenario 2*. We take the bands to have a similar shape, so that there is correlation built into the variables ${\xi }_{m}(\overrightarrow{p})$, ${\xi }_{n}(\overrightarrow{p})$. As displayed in Fig. 3, a simplified symmetric two-band structure is taken: *ε*_*g***k**_ = *ħ* ^2^ **k** ^2^/2m* and *ε*_*e***k**_ = *ε*_*g***k**_ + *G* with *G* being the average band gap between two bands and m* being the effective mass of electrons. In this case, the result is

8 $$\frac{{\rm{Re}}{\varepsilon }_{xy}}{{\rm{Im}}{\varepsilon }_{xx}}\sim \frac{4{{\rm{\Delta }}}^{2}}{{\omega }^{2}-{G}^{2}}\,{I}_{s}.$$

It is assumed that Δ_*m*_ ≈ Δ_*n*_ ≡ Δ. Intraband theories typically give ${\theta }_{K}\sim {({\rm{\Delta }}/\omega )}^{2}\sim {10}^{-8}$, which is smaller. When $\omega \mathop{ > }\limits_{ \tilde {}}G$, the result (8) predicts that *θ*_*K*_ can be highly enhanced due to a resonance effect. For instance, if *G* = 0.9*ω*, *θ*_*K*_ will be one order of magnitude larger than what is predicted by one-band theory, ${\theta }_{K}\sim {({\rm{\Delta }}/\omega )}^{2}$, in good agreement with experiment.

## Multiple Chiralities

These order-of-magnitude considerations all assume that *θ*_*K*_ does not vanish by symmetry, which of course can happen if the angular integral in Eq. (5) vanishes: *I*_*s*_ = 0. Resolving this question amounts to a symmetry analysis of the factor ${J}_{x}^{(mn)}\,(\overrightarrow{p})\,{J}_{y}^{(mn)}\,(-\overrightarrow{p})\,[{\rm{\Delta }}\,(m,\overrightarrow{p})\,{{\rm{\Delta }}}^{\ast }\,(n,\overrightarrow{p})]$ for each pair of bands that are separated by the laser photon energy 0.8 eV. Both the real and the imaginary part of $[{\rm{\Delta }}\,(m,\overrightarrow{p})\,{{\rm{\Delta }}}^{\ast }\,(n,\overrightarrow{p})]$ contribute to *θ*_*K*_. The product *J*_*x*_ *J*_*y*_ transforms according to the *E*_2*g*_ representation of *D*_6*h*_. The representation of $[{\rm{\Delta }}\,(m,\overrightarrow{p})\,{{\rm{\Delta }}}^{\ast }\,(n,\overrightarrow{p})]$ is in general reducible, and if we define it as Γ_Δ_, then the integral vanishes if and only if the *E*_2*g*_ × Γ_Δ_ representation does not contain the *A*_1*g*_ (identity) representation. *θ*_*K*_ itself will vanish if and only if the integral in Eq. (5) vanishes for every pair of bands. Again we will use the example that ${\rm{\Delta }}\,(m,\overrightarrow{p})$ and ${\rm{\Delta }}\,(n,\overrightarrow{p})$ both transform as the *E*_1*g*_ representation, but the considerations apply to all representations. For this case we have essentially two possibilities. The first is ${\rm{\Delta }}\,(m,\overrightarrow{p})\,{{\rm{\Delta }}}^{\ast }\,(n,\overrightarrow{p})\sim {p}_{z}^{2}\,({p}_{x}+i{p}_{y})\,{({p}_{x}+i{p}_{y})}^{\ast }={p}_{z}^{2}\,({p}_{x}^{2}+{p}_{y}^{2})$, and we find Γ_Δ_ = *A*_1*g*_ and since *E*_2*g*_ × *A*_1*g*_ = *E*_2*g*_, *I*_*s*_ = 0 and there is no contribution to *θ*_*K*_ for a pair of bands both of which are of the (*p*_*x*_ + *ip*_*y*_) type. The second case is ${\rm{\Delta }}\,(m,\overrightarrow{p})\,{{\rm{\Delta }}}^{\ast }\,(n,\overrightarrow{p})\sim {p}_{z}^{2}\,({p}_{x}+i{p}_{y})\,{({p}_{x}-i{p}_{y})}^{\ast }={p}_{z}^{2}\,({p}_{x}^{2}-{p}_{y}^{2}+2i{p}_{x}{p}_{y})$, which gives Γ_Δ_ = *E*_2*g*_. Since *E*_2*g*_ × *E*_2*g*_ = *A*_1*g*_ + *A*_2*g*_ + *E*_2*g*_, *I*_*s*_ ≠ 0 a pair of this type can contribute to nonzero *θ*_*K*_. Thus, continuing the analogy with the Ising model, each band being of the ± type, we see that a “ferromagnetic” state with all bands of the + type or all bands being of the - type does not lead to a Kerr rotation. We must have a mixture of “+” and “−” gaps on different bands. Qualitatively, we may perhaps think of the Kerr rotation as the creation of a broken pair that must be able to absorb both the energy and the angular momentum of the photon - this can only occur if the chirality of the two gaps involved is different [see Fig. 2(b) for a schematic plot].

## Conclusion

The observation of a nonzero Kerr rotation in UPt_3_ is seen to have a multitude of consequences, going well beyond the fact that it demands that there must be time-reversal symmetry breaking. There must also exist a pairing field in a completely filled or a completely empty band, since the photon energy exceeds the bandwidth. The observation of this pairing field is novel: in fact we believe it is the first time that it has been observed in experiments in a band that is very distant in energy from the Fermi energy. The existence of this pairing field is also not enough to explain the phenomenon: there must be a subtle pattern of relative symmetry between the bands involved, which gives additional interest to the system. There must be superconductivity with opposite chiralities *p*_*z*_(*p*_*x*_ + *ip*_*y*_) and *p*_*z*_(*p*_*x*_ − *ip*_*y*_) coexisting in the same sample. This raises many interesting questions for the topology of the electronic states. We expect that both the theory of Majorana fermions in the vortex cores and that of the protected surface states must be generalized to accommodate this additional complexity.
